# CYP1A2 suppresses hepatocellular carcinoma through antagonizing HGF/MET signaling

**DOI:** 10.7150/thno.49368

**Published:** 2021-01-01

**Authors:** Jianqing Yu, Xianfeng Xia, Yujuan Dong, Zhongqin Gong, Gang Li, George Gong Chen, Paul Bo San Lai

**Affiliations:** 1 Department of Surgery, Faculty of Medicine, The Chinese University of Hong Kong, Prince of Wales Hospital, The Chinese University of Hong Kong, Hong Kong, China.; 2 Department of Endoscopy, State Key Laboratory of Oncology in South China, Sun Yat-sen University Cancer Center, Guangzhou 510000, China.; 3 Department of Orthopedics and Traumatology, Faculty of Medicine, The Chinese University of Hong Kong, Prince of Wales Hospital, The Chinese University of Hong Kong, Hong Kong, China.; 4 Department of Otorhinolaryngology, Head and Neck Surgery, Faculty of Medicine, The Chinese University of Hong Kong, Prince of Wales Hospital, The Chinese University of Hong Kong, Hong Kong, China.; 5 Shenzhen Research Institute, The Chinese University of Hong Kong, Shenzhen, Guangdong, China.

**Keywords:** CYP1A2, HGF/MET signaling, hepatocellular carcinoma, HIF-1α, prognosis

## Abstract

**Rationale:** Hyperactivation of HGF/MET signaling pathway is a critical driver in liver tumorigenesis. Cytochrome P450 1A2 (CYP1A2) was significantly down-regulated in hepatocellular carcinoma (HCC). However, little is explored about its tumor suppressive role in HCC. In this study, we examined the functional mechanisms and clinical implication of CYP1A2 in HCC.

**Methods:** The clinical impact of CYP1A2 was evaluated in HCC patients in Hong Kong cohort. The biological functions of CYP1A2 were investigated *in vitro* and *in vivo*. A series of biochemical experiments including Western blot assay, immunohistochemistry, quantitative reverse transcription-polymerase chain reaction, and Co-immunoprecipitation assay were conducted.

**Results:** CYP1A2 expression was prominently silenced in HCC tumor tissues and the high expression of CYP1A2 was significantly correlated with lower AFP level, less vascular invasion, and better tumor-free survival in local cohort of HCC patients. The overexpression of CYP1A2 inhibited HCC cell viability and clonogenicity, reduced cell migration and invasion abilities *in vitro*, and suppressed tumorigenicity *in vivo*, whereas CYP1A2 knockdown exhibited the opposite effects. CYP1A2 significantly hindered HGF/MET signaling and Matrix metalloproteinases (MMPs) expression in HCC cells. Mechanically, CYP1A2 decreased HGF level and diminished HIF-1α expression, both of which are recognized as key regulators of MET activation. As the transcriptional activator of MET, HIF-1α was identified as a binding partner of CYP1A2. Direct binding of CYP1A2 with HIF-1α induced ubiquitin-mediated degradation of HIF-1α, inhibiting HIF-1α-mediated transcriptions.

**Conclusions:** In conclusion, our results have identified CYP1A2 as a novel antagonist of HGF/MET signaling, and CYP1A2 may serve as an independent new biomarker for the prognosis of HCC patients.

## Introduction

Hepatocellular carcinoma (HCC) is the commonest primary liver cancer and ranks the fourth cause of cancer death in 2018 1. As a clinical and biologically heterogeneous cancer with both increasing incident and mortality, it is vital to identify molecules in the pathogenesis of HCC for effective treatments and prevention. Hyperactivation of hepatocyte growth factor/c-mesenchymal-epithelial transition factor (HGF/MET) signaling contributes to tumor formation, development, maintenance, and metastasis. In HCC, the expression of MET is increased in 25% to 80% patients 2-4. Notably, a high amount of HGF and MET indicates poor differentiation and negative prognoses 5, 6. As the only acknowledged high-affinity receptor of HGF, oncogene MET modulates receptor tyrosine kinases (RTKs) and triggers a sequence of biological effect to assist proliferation and invasion of tumor cells 7, 8. In majority types of cancers, MET is transcriptionally modulated by intratumoral hypoxia, ligand-independent activation or increased susceptibility to HGF 9, and antagonized by some molecules such as interferon-induced protein 44-like (IFI44L) and angiopoietin-like protein 1 (ANGPTL1) 10, 11. However, the detailed molecular pathway responsible for suppression of HGF/MET axis in HCC is unknown.

Cytochrome P450 1A2 (CYP1A2) belongs to cytochrome P450 superfamily and is predominantly expressed in the liver. The gene CYP1A2 localizes on chromosome 15q24.1 and spans 7762 base pair of genomic sequence. The loss of CYP1A2 is associated with fast-growing malignancy and its suppression has been identified in approximately 90% HCC patients 12, 13. Importantly, after analyzing genome-wide gene-expression pattern and variable-selection procedure on both tumoral and non-tumoral tissues, Tanaka *et al.* indicated CYP1A2 as an independent predictor for post-surgical recurrence on early-staged HCC patients 14. The result was confirmed in another study showing that the majority of HCV-related HCC patients with low expression of CYP1A2 suffered from post-operative recurrence, owing to lack of protective effort on hepatocytes from the CYP1A2 15. Previous studies implied that CYP1A2 metabolites such as 17β-estradiol (E2) and cis-nerolidol could inhibit proliferation and promoting apoptosis 16, 17. However, the knowledge of CYP1A2 in HCC is limited, and the direct role of CYP1A2 in HCC progression and its signaling regulation remains unclear. In this study, we have comprehensively provided experimental based evidence to support CYP1A2 as a pivotal tumor suppressor and demonstrated a novel antagonist of HGF/MET signaling in HCC. We also explored the underlying regulation of CYP1A2 and illustrated the clinical impact of CYP1A2 in a cohort of local HCC patients.

## Materials and Methods

### HCC tissue samples

HCC tissue specimens were collected during surgery in the Prince of Wales Hospital, the Chinese University of Hong Kong, from 2007 to 2011. The study was approved by the Joint Chinese University of Hong Kong-New Territories East Cluster Clinical Research Governance and Management committee, and written informed consent was acquired from patients before the operation.

### Cell lines and cell cultures

Human HCC cell lines, Huh7, HepG2, PLC/PRF/5 were purchased from American Type Culture Collection (ATCC, Manassas, VA), and SMMC-7721 was provided by the Cell Bank of Type Culture Collection of Chinese Academy of Sciences (Shanghai, China). The Huh7, HepG2, PLC/PRF/5 were maintained in DMEM medium, and SMMC-7721 was grown in RPMI 1640. Both media were supplemented with 10% FBS (Thermo Fisher Scientific, Waltham, MA) and Antibiotic-Antimycotic (100X) (Gibco, Thermo Fisher Scientific).

### Chemicals

HGF and PHA-665752 were obtained from Santa Cruz Biotechnology (Santa Cruz, CA), LY294002 was obtained from Cayman Chemical (Ann Arbor, MI), and cycloheximide was ordered from Abcam (Cambridge, United Kingdom). Cells were starved with serum-free medium overnight before administration of HGF, PHA-665752, LY294002, or cycloheximide.

### Plasmids, lentivirus generation, and cell infection

CYP1A2-pcDNA3.1(+) was a gift from Jianwei REN 16. For stable overexpression of CYP1A2, CYP1A2-pcDNA3.1 was transfected by lipofectamine 3000 and selected by G418 (Gibco). The empty vector was served as scramble control. For knockdown of CYP1A2 in Huh7 and SMMC-7721 cells, shRNA targeting CYP1A2 was designed from RNAi Consortium library (www.broadinstitute.org/rnai/public), and the target sequence was CAAGGGACACAACGCTGAATG. Oligos for hairpin construction were subcloned into pLKO.1 (Addgene). Lentivirus was produced by co-transfection of pLKO.1, psPAX2, and pMD2.G, and infected cells were screened by puromycin (Invitrogen, Carlsbad, CA). The oligo sequences were shown in Table S1. For siRNA experiments, cells were transfected with HIF-1α siRNA (Santa Cruz) or scrambled siRNA as a control for 24 h.

### Immunohistochemistry

The performance was based on the previous description 18 and the scoring system (IRS) method was applied to evaluate the immunohistochemical results according to the stated grading system. The primary antibodies were shown in Table S2.

### *In vitro* cell functional assays

Cell viability was evaluated by the 3-(4,5-Dimethylthiazol-2-yl)-2,5-diphenyltetrazolium bromide (MTT) assay. HCC cells (2 × 10^3^/well) were cultured in 96-well plates for different time-points. For colony formation assay, cells (1 × 10^3^/well) were seeded in 6-well plates. Cells were stained with 0.1% crystal violet after 10-14 days, and colonies were counted with ImageJ. For wound healing assay, the protocol was followed in order to reduce the effect of cell proliferation 19. Briefly, confluent cells in 6-well plates were starved with serum-free DMEM medium overnight and scratched with sterilized tips. The cells were then cultured in DMEM with 0.5% FBS and incubated for 24 h. For the cell invasion assay, cells (5 × 10^4^/well) were cultured with the serum-free medium in the upper chamber (8 μm pore size) (Corning, Wiesbaden, Germany) coated with 20% Matrigel (Corning). DMEM containing 10% FBS was supplied in the lower chamber as a chemoattractant. After 72 h, cells that have invaded through the upper chamber were fixed in methanol and stained with 0.1% crystal violet.

### *In vivo* tumor xenograft assay

CYP1A2-overexpressed PLC/PRF/5 or CYP1A2-knockdown Huh7 and the respective control cells (2.5×10^6^ cells in 100 μL phosphate-buffered saline) were subcutaneously injected into the back of nude mice (4-6 weeks) to form xenograft. The tumor volume was evaluated by the formula (length × width^2^)/2. The tumors were embedded in paraffin for IHC. All experimental procedures were approved by the Animal Ethics Committee of the Chinese University of Hong Kong, and the humane endpoints are in accordance with the guidelines for the welfare and use of animals in cancer research 20.

### RNA isolation and quantitative real-time PCR

Total RNA was extracted by TRIzol™ Reagent (Invitrogen), and the reverse-transcription was performed by EvoScript Universal cDNA Master (Roche, Basel, Switzerland). Genes of interest were examined by SYBR Premix Ex Taq (TAKARA, Japan) on the Quantstudio™ 12k Flex Real-time PCR system (Applied Biosystems). The expression of target genes was normalized to GAPDH, and the delta-delta-CT method was used to quantify results. The primer sequences are listed in Table S1.

### Western blotting

The procedures of protein extraction and concertation measurement were mentioned previously 18. The used antibodies are listed in Table S2.

### Co-immunoprecipitation assay

Total protein from Huh7 cells was extracted in ice-cold RIPA buffer (150 mM NaCl, 0.5% sodium deoxycholate, 1% NP-40, 0.1% SDS, 1 mM EDTA, 50 mM Tris, pH = 7.4) supplemented with protease inhibitor cocktail (Roche). Cell lysates were collected and immunoprecipitated with either 2 μg indicated antibodies or anti-IgG at 4 °C overnight with gentle rotation. Protein A/G PLUS-Agarose (sc-2003, Santa Cruz) was used to pull down the antibodies. After washing with PBS three times, immunoprecipitated proteins were added with a 2X loading buffer and boiled at 95 °C for 5 min.

### Ubiquitination assay

PLC/PRF/5 and Huh7 cells stably transfected with CYP1A2 expression vector or vehicle control were incubated in the presence or absence of 10 μM MG132 (Sigma-Aldrich, St. Louis, MI) for 12 h. Total proteins were extracted by RIPA buffer, and anti-HIF-1α or anti-IgG was used for immunoprecipitation. Eluates were subjected to Western blotting assay to evaluate ubiquitination level with anti-ubiquitin antibody (ab7254, Abcam).

### Immunofluorescent Staining

Cells were fixed by 4% paraformaldehyde (4% PFA/PBS) and permeabilized with 0.1% Triton X-100 at room temperature for 15 min. After blocking with 0.1% FBS, cells were blocked and immunolabeled with Anti-CYP1A2 (sc-53241) and Anti-HIF-1α (ab2185) at 4 °C overnight. On the following day, fluorescence-conjugated secondary antibodies, Alexa Fluor 488-conjugated donkey anti-rabbit and Alexa Fluor 568-conjugated donkey anti-mouse (both from Thermo Fisher), were stained at room temperature for 2 h. Nuclei were counterstained with DAPI (Invitrogen) for observation.

### Analysis of gene signatures

To identify the correlation between CYP1A2 and HIF-1α in online database, GEPIA (http://gepia.cancer-pku.cn/) was used for further investigation. The raw data of CYP1A2 mRNA expression in different clinical grades were downloaded from the HCC TCGA dataset in TSVdb (http://www.tsvdb.com/plot.html).

### Statistical analysis

Experiments were performed in triplicates, and data were plotted as mean ± standard deviation. The student's *t*-test or Mann-Whitney *U* test was applied to compare the means between two groups and one-way ANOVA with the Dunn's post-test was for multiple groups. Two-way analysis of variance was performed for xenograft growth curves evaluation. The high- and low expression of CYP1A2 was defined by Receiver Operating Characteristic (ROC) curve. The effect of CYP1A2 on survival was performed by the Kaplan-Meier survival curve and analyzed by the log-rank test. OS was calculated as the time from the surgery to death or the last follow-up date. TFS was defined as the days from surgery to the first-time recurrence of HCC. Clinical pathological features were analyzed by Pearson's chi-squared test or Fisher's exact test. Statistical analyses were conducted by Graph Pad Prism version 8.0.2 (GraphPad, La Jolla, CA), and *P* < 0.05 was regarded as statistically significant.

## Results

### CYP1A2 is frequently silenced in primary HCC tumors

**Figure 1:**
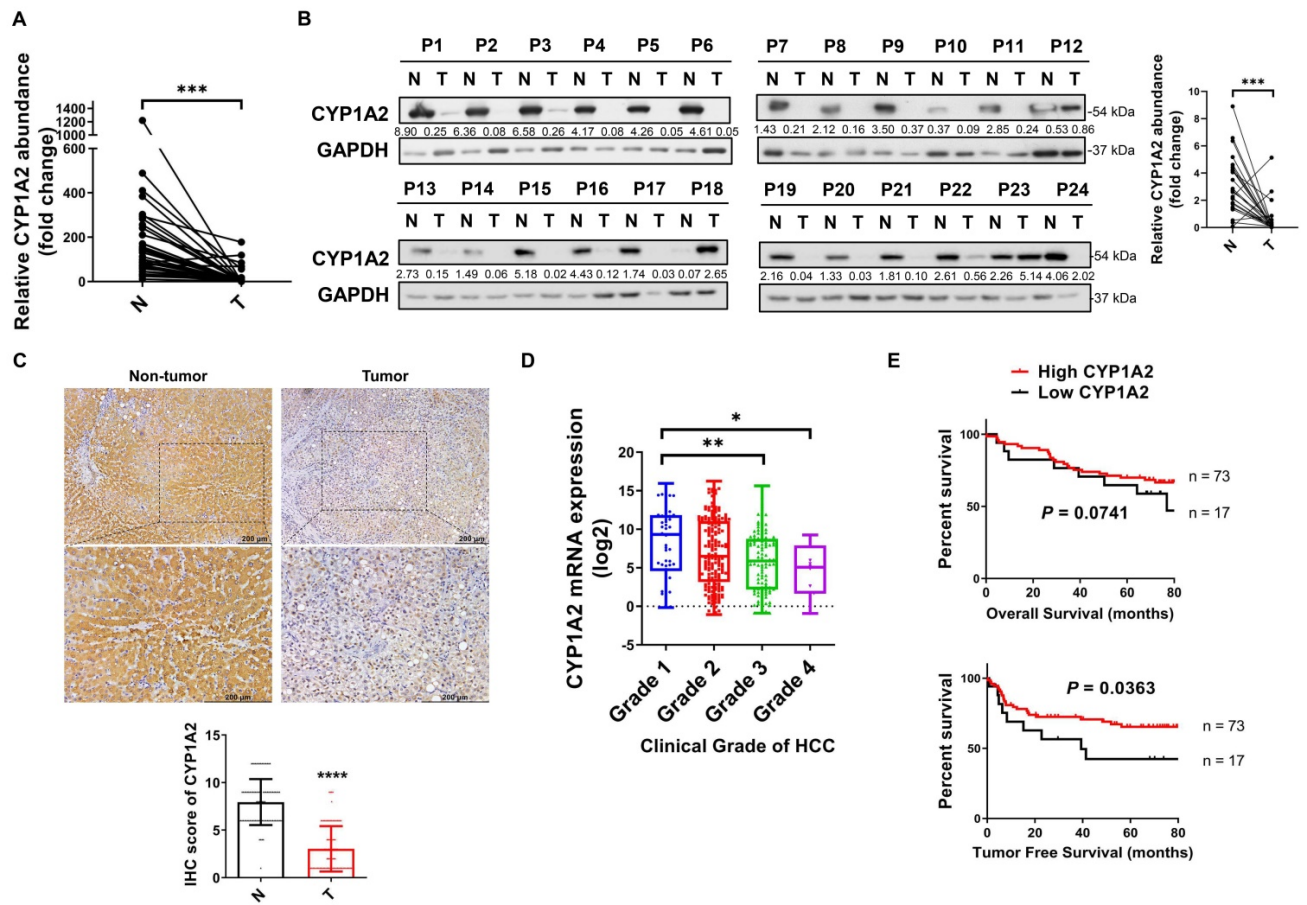
** CYP1A2 is significantly downregulated in HCC. (A)** Real-time PCR was used to determine CYP1A2 mRNA in 37 pairs of the randomly selected primary tumor and matched adjacent non-tumor tissues from HCC patients (****P < 0.0001). Expression data were normalized to GAPDH. **(B)** CYP1A2 protein in 24 pairs of randomly selected HCC tumor and adjacent non-tumor tissues were evaluated by Western blot analysis. The number below the blots indicated the ratio of targeted protein to GAPDH. **(C)** IHC staining of CYP1A2 in 90 pairs of primary HCC tissues and adjacent non-tumor tissues (****P < 0.0001). Photomicrographs showed the representative IHC staining of CYP1A2 in HCC tissues under low-power and high-power magnification. **(D)** The mRNA expression of CYP1A2 in different clinical grades of TCGA HCC patients (*P < 0.05, **P < 0.01). Grade 1: n = 52; Grade 2: n = 165; Grade 3: n = 108; Grade 4: n = 12. **(E)** Kaplan-Meier survival analysis displayed patients with HCC with high CYP1A2 mRNA expression had better TFS than those with low CYP1A2 from the Hong Kong cohort. The high- and low expression of CYP1A2 was defined by Receiver Operating Characteristic (ROC) curve. For statistical analysis in (A), (C) and (D), data were expressed as mean ± standard deviation. N, adjacent non-tumor tissues; T, tumor tissues.

To identify the dysregulated CYP1A2 expression, we evaluated both mRNA and protein expression status in HCC tissues and the paired non-tumor tissues. Compared to the predominant expression in the non-tumor tissues, the CYP1A2 mRNA level was markedly decreased (by 11.3-fold on average) in HCC samples (Figure 1A). The reduced CYP1A2 mRNA was further confirmed in the TCGA dataset consisting of 340 patients with HCC (Figure S1). In line with down-regulated CYP1A2 mRNA, the reduction of CYP1A2 protein (5.5-fold) was demonstrated by Western blotting assay in the 24 randomly selected HCC cases (Figure 1B). In addition, IHC staining showed a significant weak expression of CYP1A2, with 2.6-fold suppression, in HCC tissues compared with adjacent non-tumor tissues (Figure 1C). Therefore, the results of all these assays support the downregulation of CYP1A2 in HCC.

### CYP1A2 is associated with survival in HCC patients

To determine the clinical significance of CYP1A2 in HCC patients, we firstly analyzed the data from the TCGA database. The results showed that patients with a decreased level of CYP1A2 exhibited the greater clinical grade of HCC (Figure 1D), but the expression was not associated with clinical pathological stages (Figure S2). Furthermore, we measured CYP1A2 mRNA expression in a cohort of 90 HCC patients in our tissue bank and stratified into high and low expression groups. It was demonstrated that the expression of CYP1A2 was significantly associated with the serum alpha-fetoprotein (AFP) level and vascular invasion (Table 1). Kaplan-Meier analysis revealed that the elevated CYP1A2 expression was statistically correlated with longer tumor-free survival (TFS) but not overall survival (OS) (Figure 1E). Consistent with our result, CYP1A2 overexpression extended survival in patients of the HCCDB database (Figure S3).

### CYP1A2 suppresses HCC cell proliferation, migration, and invasion *in vitro* and tumorigenesis *in vivo*

**Figure 2:**
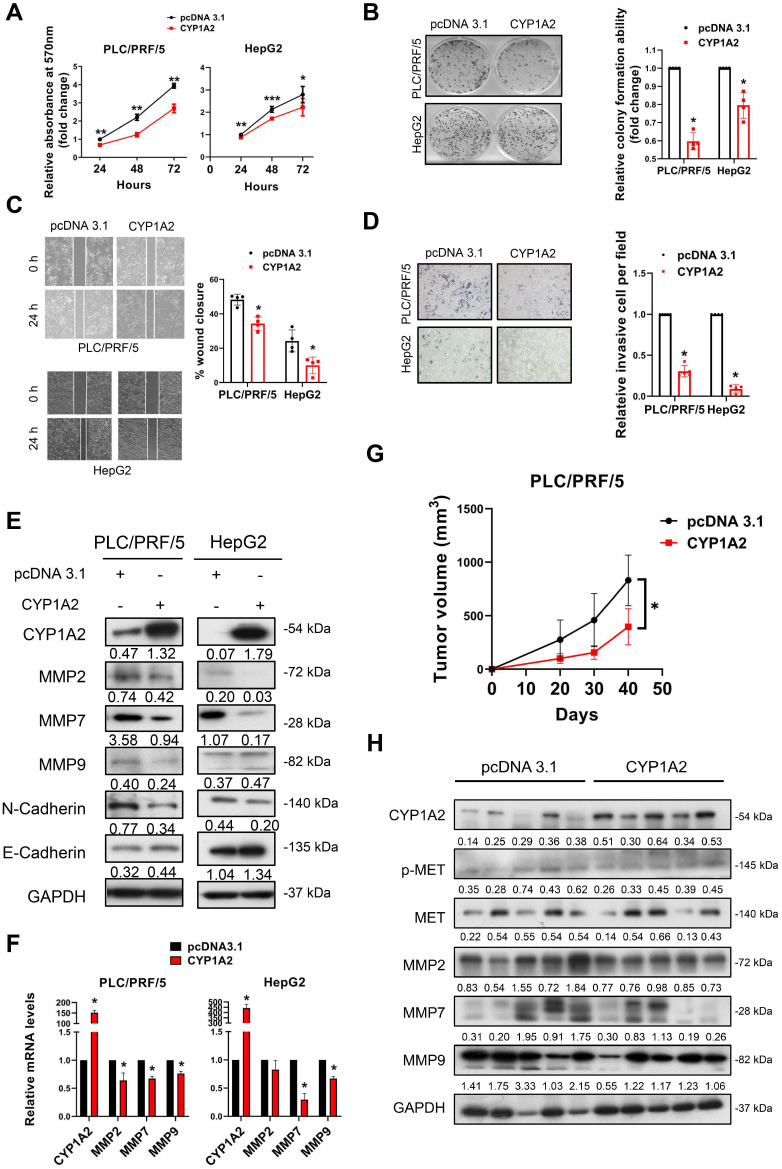
** CYP1A2 suppresses HCC cell growth *in vitro* and *in vivo*. (A)** MTT assay was used to evaluate the effects of CYP1A2 of cell proliferation in HCC cells (*P < 0.05, **P < 0.01). **(B)** Colony formation assay on CYP1A2-overexpressed cells compared with the scramble control in a fold-change manner (*P < 0.05). The numbers of relative colonies were 0.60 ± 0.05 and 0.80 ± 0.07 in the CYP1A2-overexpressed PLC/PRF/5 and HepG2 cells, respectively. **(C)** Overexpression of CYP1A2 inhibited cell migration as displayed by wound-healing assay (*P < 0.05). **(D)** Matrigel invasion assay showed CYP1A2 significantly inhibited cell invasion capability (*P < 0.05). The relative invasive cells were 0.30 ± 0.07 and 0.09 ± 0.05 in CYP1A2-overexpressed PLC/PRF/5 and HepG2 cells, respectively. **(E)** CYP1A2 decreased the expression of mesenchymal marker (N-cadherin) and MMPs while increased the expression of epithelial markers (E-cadherin) measured by Western blot. The number below the blots indicated the ratio of targeted protein to GAPDH. **(F)** CYP1A2 inhibited the expression of MMPs mRNA as shown by Real-time PCR (*P < 0.05). **(G)** Growth curves of xenograft tumors (N = 5 for each group) formed by the subcutaneous injection of CYP1A2-overexpressed PLC/PRF/5 cells and scramble control (*P < 0.05). **(H)** Western blot assay illustrated the decreased expression of MET as well as MMPs in the xenograft tumors. For experiments (E) and (H), the number below the blots indicated the ratio of targeted protein to GAPDH. All data were presented as mean ± standard deviation.

The significant reduction of CYP1A2 prompted us to examine its potential tumor inhibitory role in HCC. Therefore, we performed gain- and loss-of-function assays on CYP1A2. Firstly, we overexpressed CYP1A2 in PLC/PRF/5 and HepG2 cells as their endogenous levels of CYP1A2 were low (Figure S4). We firstly evaluated the effect of CYP1A2 on cell growth, and it was shown that CYP1A2 evidently decreased the cell viability (Figure 2A) and colony formation ability (Figure 2B) in both HCC cells. Next, we examined the cell invasiveness by wound-healing assay and transwell invasion assay. The result indicated that CYP1A2 overexpression markedly impaired cell migration (Figure 2C) and invasion abilities (Figure 2D). It is well accepted that epithelial-to-mesenchymal transition (EMT) programs are highly associated with enhanced migration and invasion 21. Apart from the well-known EMT markers, several matrix metalloproteases (MMPs) have been implicated in EMT-regulated tumor progression 22 and highly upregulated in the TCGA-LIHC dataset (Figure S5). The Western blotting assay illustrated the down-regulated expression of mesenchymal marker (N-Cadherin) and MMPs, along with the increased level of the epithelial marker E-cadherin (Figure 2E) in CYP1A2-overexpressed PLC/PRF/5. Consistently, the mRNA levels of MMP2, MMP7, and MMP9 were also significantly decreased with CYP1A2 overexpression compared with the scrambled control (Figure 2F). In light of the *in vitro* findings, we assessed the effect of CYP1A2 on tumorigenesis *in vivo*. Results showed that CYP1A2 overexpression notably shrank the xenograft tumor volume (Figure 2G). The reduction of the tumor volume by CYP1A2 was supported by the deactivated MET (Figure 2H) and decreased expression of MMPs in xenograft tumor tissues (Figure 2H). These findings demonstrated that CYP1A2 functioned to hinder the progression of HCC both *in vitro* and *in vivo*.

### CYP1A2 knockdown promotes HCC cell progression *in vitro* and tumorigenicity *in vivo*

**Figure 3:**
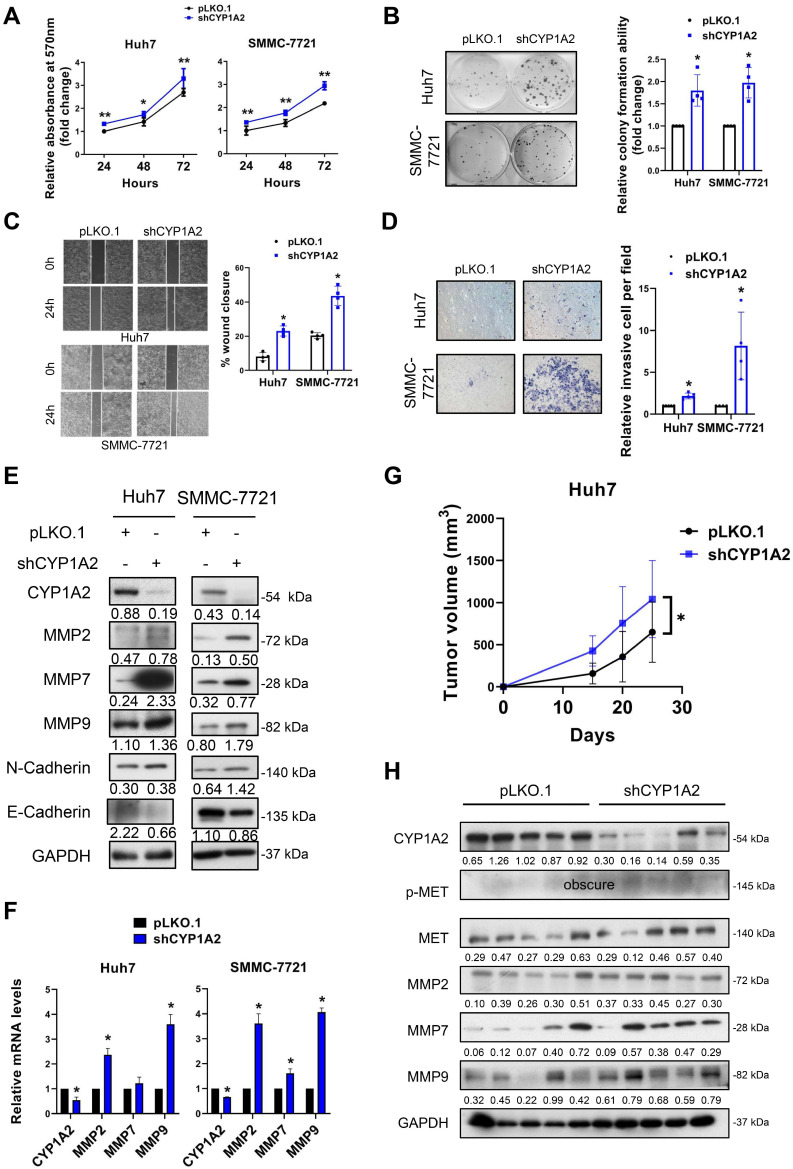
** Loss of CYP1A2 promotes HCC development *in vitro* and *in vivo*. (A)** The knockdown of CYP1A2 increased HCC cell proliferation as shown by MTT assay (*P < 0.05, **P < 0.01). **(B)** The colony formation ability was enhanced in CYP1A2-knockdown HCC cells (*P < 0.05). The numbers of relative colonies were 1.80 ± 0.35 and 1.97 ± 0.34 in the CYP1A2-deficient Huh7 and SMMC-7721 cells, respectively. **(C)** Loss of CYP1A2 elevated migratory abilities in HCC cells as shown by wound-healing assay (*P < 0.05). **(D)** The CYP1A2-deficient HCC cells exhibited stronger invasive abilities than the scramble control (**P < 0.01). The relative invasive cells were 2.17 ± 0.35 and 8.15 ± 4.05 in CYP1A2-knockdown Huh7 and SMMC-7721 cells, respectively. **(E)** The CYP1A2-knockdown HCC cells increased the protein expression of MMPs. **(F)** The mRNA expression of MMPs were increased in CYP1A2-knockdown HCC cells (*P < 0.05). **(G)** Growth curves displayed the tumor volume after subcutaneous injection of CYP1A2-knockdown Huh7 cells compared with scramble control (N = 5 for each group). Loss of CYP1A2 increased HCC tumor growth *in vivo* (*P < 0.05). **(H)** The upregulation of MET and MMPs in xenograft tumors was demonstrated by the Western blot assay. For experiments (E) and (H), the number below the blots represented the ratio of targeted protein to GAPDH. All data were displayed as mean ± standard deviation.

To determine the loss of function of CYP1A2, we silenced CYP1A2 in Huh7 and SSMC-7721 cells by lentivirus shRNA plasmids. Compared to scramble control, the MTT assays revealed that the knockdown of CYP1A2 significantly enhanced the proliferation of Huh7 and SMMC-7721 cells (Figure 3A). Similarly, a greater number of colonies were observed in both CYP1A2-deficient HCC cells, indicating the CYP1A2 depletion markedly promoted colony formation abilities (Figure 3B). Besides, the accelerated wound closures suggested that the loss of CYP1A2 significantly elevated the migration abilities in HCC cells (Figure 3C). The invasion capacity was also markedly stimulated in the CYP1A2 knockdown Huh7 cells (Figure 3D). The loss of CYP1A2 patently up-regulated the protein expression of MMPs, whereas cadherins remained unchanged (Figure 3E), suggesting that the CYP1A2-silenced HCC progression was MMPs-related. Consistently, the marked enhancement of mRNA levels was observed on MMP2 and MMP9 (Figure 3F), but not on N- and E-Cadherin. Additionally, subcutaneous xenograft models suggested the increased tumorigenicity (Figure 3G) and up-regulated MET and MMPs expression in xenograft tumors formed by CYP1A2-deficient HCC cells (Figure 3H). Taken together, the loss of CYP1A2 aggravated HCC progression both *in vitro* and *in vivo*.

### CYP1A2 inhibits HGF/MET signaling

**Figure 4:**
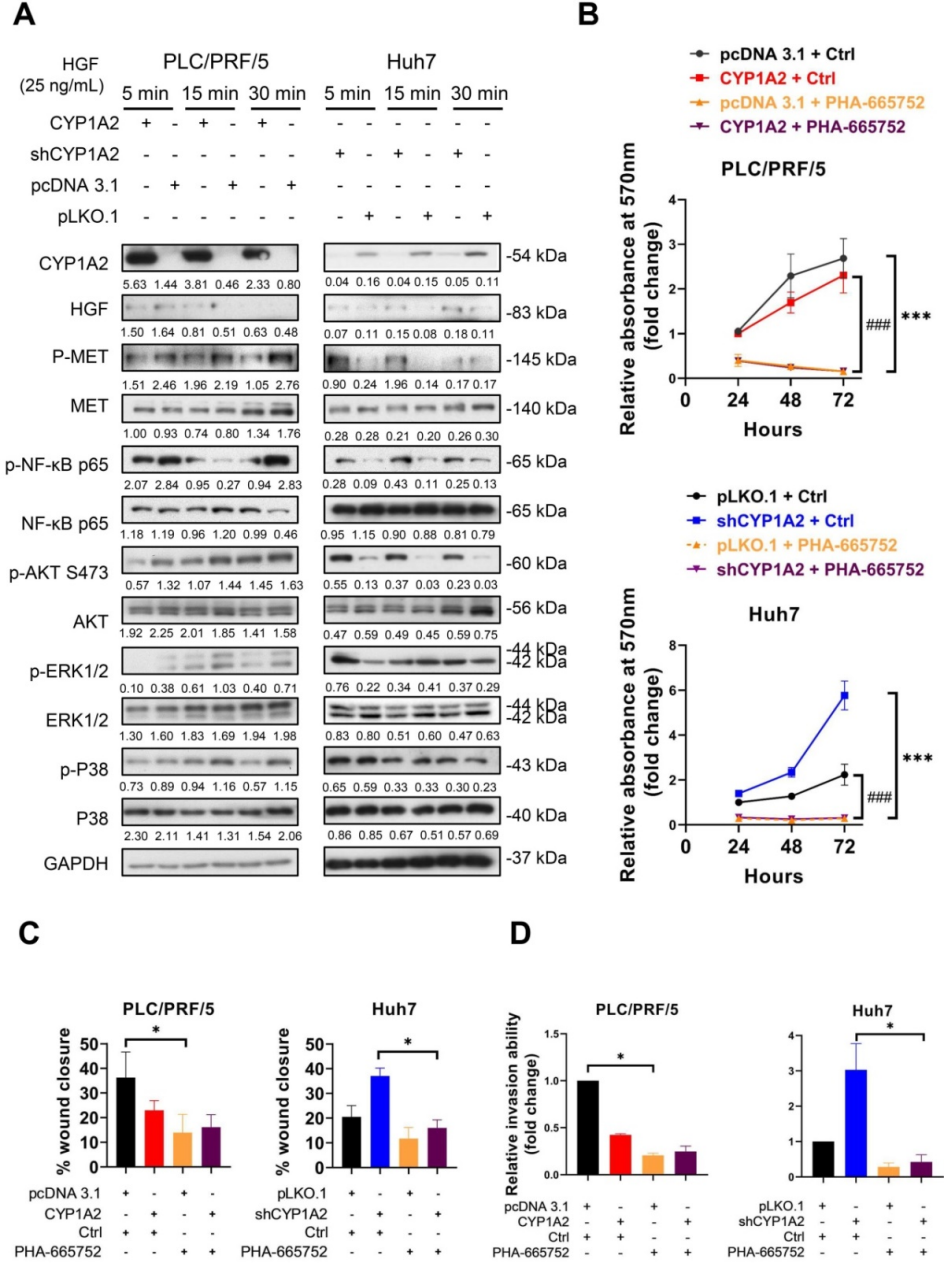
** CYP1A2 inhibits HGF-induced MET and its downstream molecules. (A)** Western blot assay illustrated the expression of HGF/MET and its downstream molecules after administration of HGF (25 ng/mL) for 5, 15, and 30 mins in CYP1A2-overexpressed PLC/PRF/5 and CYP1A2-knockdown Huh7 The number below the blots indicated the ratio of targeted protein to GAPDH. **(B)** MTT assay showed that PHA-665752 (10 µM) significantly inhibited cell proliferation in CYP1A2-absent HCC cells (***P < 0.001, ###* P* < 0.001) (*: Scramble+Ctrl versus Scramble+PHA-665752; #: CYP1A2+Ctrl versus CYP1A2+PHA-665752 or shCYP1A2+Ctrl versus shCYP1A2+PHA-665752). **(C)** The inhibitory effect of PHA-665752 (10 µM) on HCC cell migration (*P < 0.05). PHA-665752 reduced the migration rate in CYP1A2-overexpressed PLC/PRF/5 and CYP1A2-knockdown Huh7 and their control cells. **(D)** Trans-well invasion assay indicated that PHA-665752 (10 µM) diminished HCC invasive abilities (*P < 0.05). The relative invasive cells were 0.42 ± 0.01 for “CYP1A2 + Ctrl”, 0.21 ± 0.02 for “pcDNA 3.1 + PHA-665752”, and 0.25 ± 0.06 for “CYP1A2 ± PHA-665752” in PLC/PRF/5 cells. In Huh7 cells, the relative invasive cells were 3.03 ± 0.74 for “shCYP1A2 + Ctrl”, 0.29 ± 0.11 for “pLKO,1 + PHA-665752”, and 0.43 ± 0.20 for “shCYP1A2 + PHA-665752”, respectively. All data were displayed as mean ± standard deviation. Ctrl, DMSO control.

After binding to MET, HGF mediates cell proliferation, cell motility as well as morphogenesis by stimulating a tyrosine kinase signaling cascade, and it is one of the growth factors responsible for triggering MMPs in cancers. To identify whether CYP1A2 could attenuate HGF-induced MET, stably transfected HCC cells were starved with serum-free DMEM overnight and administrated of HGF (25 ng/mL or 40 ng/mL) for 5, 15, and 30 min. Western blot analysis showed that the overexpression of CYP1A2 significantly deactivated MET and its downstream signaling molecules, including NF-κB p65, AKT, ERK, and P38 (Figure 4A, Figure S6). On the other hand, the knockdown of CYP1A2 evidently activated NF-κB p65 and AKT, but the induction of ERK and P38 MAPK was hardly observed (Figure 4A, Figure S6). Since HGF/MET signaling played a critical role in HCC cell proliferation, migration, and invasion 23, we investigated whether administration of MET inhibitor could abolish these abilities in the presence or absence of CYP1A2 in HCC cells. The PHA-665752 is a selective inhibitor of MET, which exerts strong inhibitory ability in MET-high HCC cells 24. We firstly examined the anti-proliferative potential of PHA-665752 on PLC/PRF/5 and Huh7 cells. The MTT assay showed that 10 μM PHA-665752 obviously suppressed the cell proliferation at 48 and 72hrs compared to the DMSO control (Figure S7). Further investigation suggested that PHA-665752 (10 μM) significantly hindered HCC cell proliferation at different time points (Figure 4B). Besides, the migrative and invasive abilities were markedly suppressed by PHA-665752 (10 μM) when CYP1A2 was blocked in HCC cells (Figure 4C, 4D). Consistently, silencing of MET by the siRNA decreased the expression of MMP2 and MMP9, and abolished the proliferation, migration, and invasion effects in the absence of CYP1A2 in Huh7 cells (Figure S8). Collectively, the results suggested that MET signaling was stimulated in the absence of CYP1A2, and the increased proliferation, migration, and invasion capacities could be reversed by MET inhibition in HCC cells *in vitro*.

### Inhibition of PI3K/AKT/NF-κB axis suppresses HGF-induced proliferation, migration, and invasion on HCC cells

**Figure 5:**
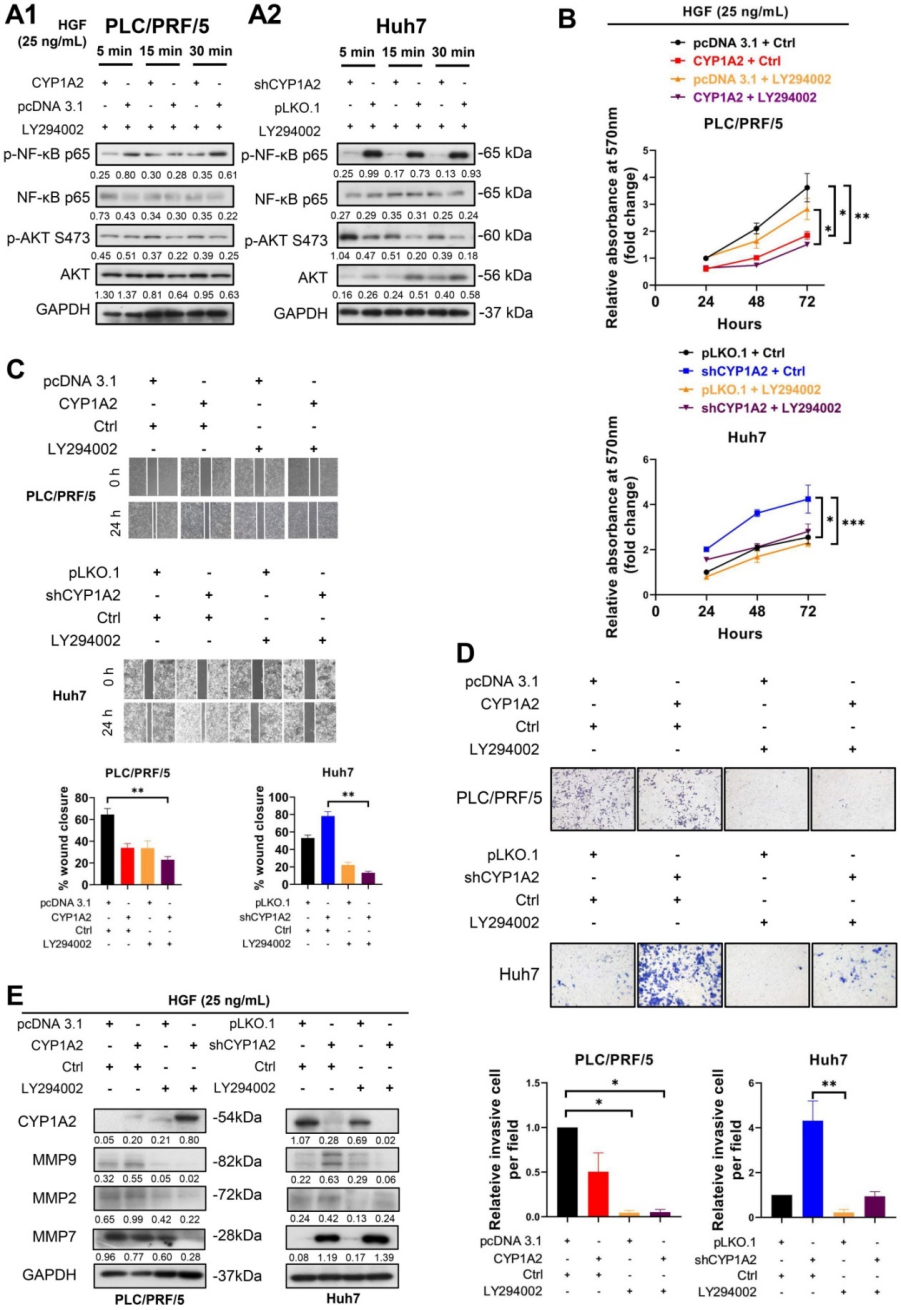
** Inhibition of PI3K/AKT/NF-κB axis reverses HGF-induced proliferation, migration, and invasion in HCC cells. (A1)** The protein expression of AKT and NF-κB after co-administration of HGF (25 ng/mL) and LY294002 (15 µM) in CYP1A2-overexpressed PLC/PRF/5 and the control cells. **(A2)** Western blot assay showed co-administration of HGF (25 ng/mL) and LY294002 (15 µM) in CYP1A2-absent Huh7 cells. **(B)** Co-administration of HGF (25 ng/mL) and LY294002 (15 µM) significantly reduced cell viability in the present or absent of CYP1A2 in HCC cells (*P < 0.05, **P < 0.01, ***P < 0.001). **(C)** LY294002 (15 µM) evidently decreased HGF-mediated cell migration as shown by wound-healing assays (**P < 0.01). **(D)** LY294002 (15 µM) evidently rescued HGF-induced cell invasion ability (*P < 0.05, **P < 0.01). The relative invasive cells were 0.50 ± 0.21 for “CYP1A2 + Ctrl”, 0.05 ± 0.02 for “pcDNA 3.1 + LY294002”, and 0.05 ± 0.03 for “CYP1A2 ± LY294002” in PLC/PRF/5 cells. In Huh7 cells, the relative invasive cells were 4.32 ± 0.88 for “shCYP1A2 + Ctrl”, 0.22 ± 0.13 for “pLKO,1 + LY294002”, and 0.94 ± 0.21 for “shCYP1A2 + LY294002”, respectively. **(E)** After administration of HGF (25 ng/mL) and LY294002 (15 µM) for 24hrs, combined treatment evidently diminished expression of MMP2 and MMP9 in both PLC/PRF/5 and Huh7 cells but had no effect on MMP7 in CYP1A2-knockdown Huh7 cells. For experiments (B), (C), (D), and (E), Ctrl represented DMSO control. For experiment (A) and (E), the number below the blots showed the ratio of targeted protein to GAPDH. All data were indicated as mean ± standard deviation. Ctrl, DMSO control.

Previous studies have demonstrated HGF-induced NF-κB activation is regulated by PI3K/AKT in gastric cancer 25, 26. Therefore, to examine whether the modulation axis was also applicable to HCC, we administrated HGF accompanied by LY294002, a potent, selective suppressor of PI3K 27, to HCC cells. Compared with the administration of HGF in Figure 4A, after co-administration of HGF and LY294002, the activated AKT was diminished in PLC/PRF/5 scramble cells (Figure 5A1). It is noticeable that the up-regulated AKT was greatly diminished, and NF-κB p65 phosphorylation was significantly reversed in the CYP1A2-knockdown Huh7 cells (Figure 5A2), confirming that NF-κB was the downstream effector of PI3K/AKT axis.

Next, we examined whether LY294002 could abolish HGF-mediated tumor growth *in vitro*. MTT assay showed LY294002 effectively hindered HGF-stimulated cell growth not only on CYP1A2-overexpressed PLC/PRF/5 cells but on CYP1A2-silenced Huh7 cells (Figure 5B). Moreover, HGF accelerated the wound healing rate more efficiently on scrambled control compared with CYP1A2-overexpressed PLC/PRF/5, while the phenomenon could be prevented from co-administration with LY294002 (Figure 5C). On the contrary, HGF significantly enhanced the migration ability in CYP1A2-knockdown Huh7 cells, but the inverse effect was observed when given LY294002 (Figure 5C). Similarly, the invasion assay illustrated HGF performed stronger invasive capability in CYP1A2-deficient HCC cells, and the impact was counteracted by LY294002 (Figure 5D). Western blot analysis illustrated that LY294002 greatly restrained HGF-induced MMP2 and MMP9 expression in the PLC/PRF/5 and Huh7 cells, but the inhibitory effect was not observed on MMP7 in Huh7 cells (Figure 5E). These results indicated that suppression of PI3K/AKT/NF-κB signaling by LY294002 could effectively inhibit the proliferation, migration as well as invasion through inhibiting MMPs in HCC.

### CYP1A2 inhibits MET signaling by diminishing HGF level and directly binding with HIF-1α

**Figure 6:**
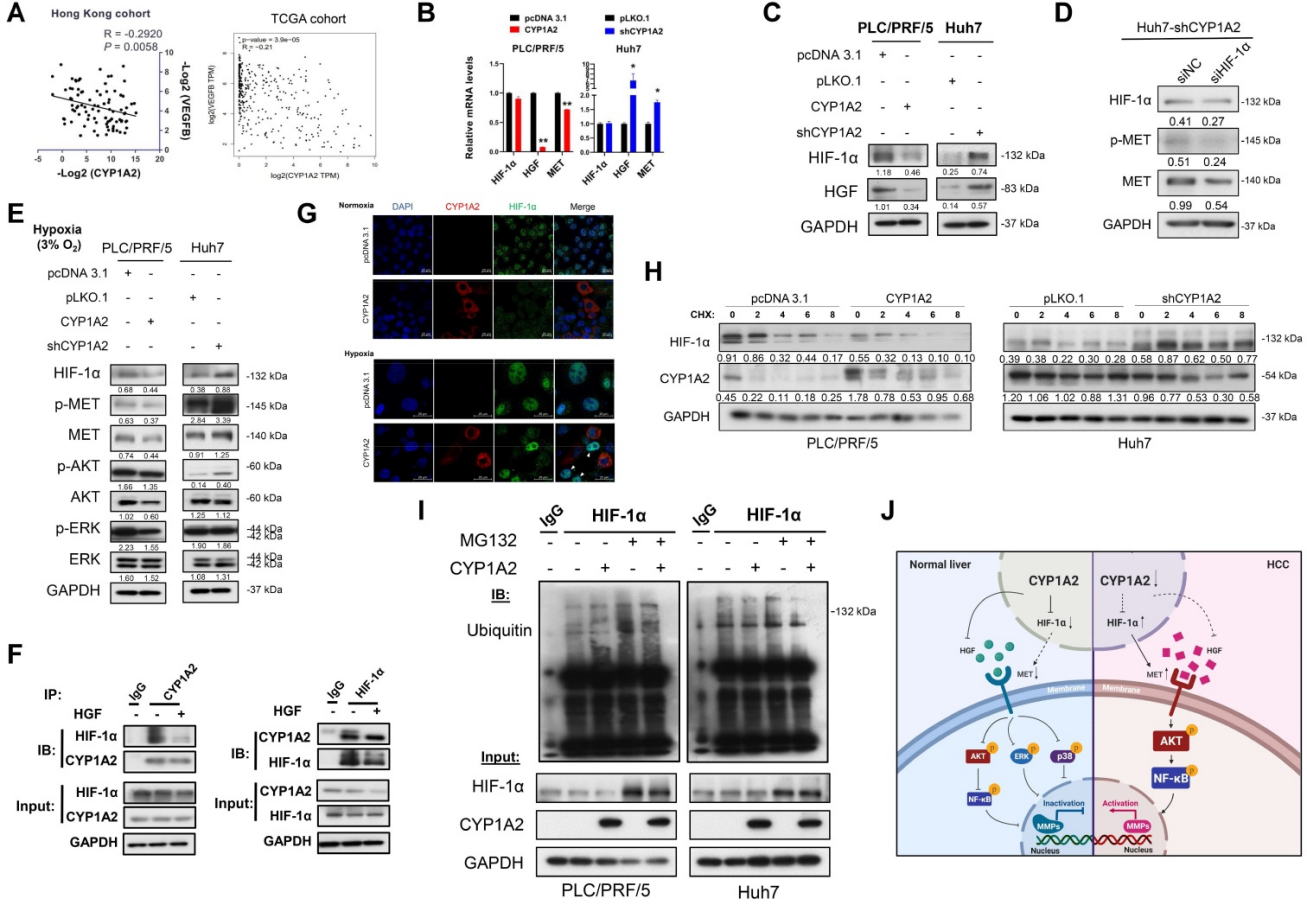
** CYP1A2 inactivates HGF/MET signaling pathway by inhibiting HGF and HIF-1α expression. (A)** Correlation between CYP1A2 mRNA and HIF-1α-targeted gene VEGFB was validated in Hong Kong HCC cohort in the Hong Kong cohort using qPCR and further confirmed in TCGA-LIHC dataset. **(B)** mRNA alternation of HIF-1α, HGF, and MET in the presence or loss of CYP1A2 in PLC/PRF/5 and Huh7 cells through qPCR analysis (*P < 0.05, **P < 0.01). **(C)** Western blot assay showed that CYP1A2 suppressed the expression of HIF-1α and HGF proteins, while CYP1A2-knockdown displayed the opposite effect. **(D)** Knockdown of HIF-1α using siRNA significantly inhibited MET protein expression. **(E)** The inhibitory effect of CYP1A2 on HIF-1α and MET signaling was confirmed under hypoxic (3% O_2_) environment. **(F)** Co-IP followed by Western blot assay indicated the direct binding between CYP1A2 and HIF-1α in Huh7 cells. **(G)** Representative images of co-localization between CYP1A2 on HIF-1α in enriched CYP1A2-overexpreesing and control PLC/PRF/5 cells by the confocal microscope. Arrowheads indicated the stronger signal of HIF-1α in the cells that absence of CYP1A2. **(H)** CYP1A2-overexpressed PLC/PRF/5 and CYP1A2-knockdown Huh7 cells were administrated with protein synthesis inhibitor cycloheximide (CHX) (100 µg/mL) for 0, 2, 4, 6, and 8 h, respectively. The loss of CYP1A2 stabilized HIF-1α expression. **(I)** MG132 (10 µM) increased the HIF-1α level by hindering its ubiquitin-regulated degradation. **(J)** Schematic diagram depicts the role of CYP1A2 in HGF/MET signaling in HCC. In brief, in normal liver, the high expression of CYP1A2 deactivates MET and its downstream molecules by inhibiting HGF and HIF-1α expression. While in HCC, the CYP1A2 is downregulated, which motivates HGF and HIF-1α, and subsequently stimulates MET/AKT/NF-κB/MMPs axis. The activation of MMPs consequently promotes the cell proliferation, migration, and invasion abilities in HCC. The schematic figure was created by the Biorender.com. For experiment (C), (D), (E), and (G), the number below the blots represented the ratio of targeted protein to GAPDH. The data in the experiment (B) were indicated as mean ± standard deviation.

As mentioned above, MET can be up-regulated by intratumoral hypoxia or increased susceptibility to HGF. It has been reported that hypoxia-inducible factor 1-alpha (HIF-1α) transcriptionally enhanced MET expression by binding to its promoter 9. We noted that there were negative correlations between CYP1A2 mRNA level and HIF-1α-targeted gene VEGFB, not only in Hong Kong cohort but also in TCGA liver hepatocellular carcinoma (LIHC) dataset (Figure 5A, Figure S9). To gain insights into whether CYP1A2 can regulate HGF and HIF-1α expression, we evaluated the effect of CYP1A2 on the expression of HGF and HIF-1α at both mRNA and protein levels. The overexpression of CYP1A2 led to a significant decreased mRNA and protein expression of HGF, while the knockdown of CYP1A2 illustrated the opposite effect (Figure 6B, C). Similar results were found at the protein level of HIF-1α; however, its mRNA expression was not obviously changed by overexpression or knockdown of CYP1A2 (Figure 6B, C). According to the previous report, HIF-1α can be modulated by PI3K/AKT axis and effectively inhibited by targeting the signaling pathway 28. Therefore, we also evaluated the HIF-1α expression after administration of LY294002 to examine whether HIF-1α was at the downstream of HGF/MET/PI3K/AKT/NF-κB axis. Western blotting assay showed that, unlike the obvious decrease of AKT and NF-κB (Figure 5A2), the protein level of HIF-1α was not significantly affected in CYP1A2-knockdown Huh7 (Figure S10). The results suggested that the HIF-1α was likely functional at the upstream of PI3K to modulate MET signaling. Next, since CYP1A2-knockdown significantly induced HIF-1α expression, we examined the effect of silencing HIF-1α on MET expression in the CYP1A2-deficient Huh7 cells. The Western blot assay showed that the HIF-1α siRNA inhibited HIF-1α expression and MET activation (Figure 6D). Since it is widely accepted that HIF-1α becomes stable upon hypoxia 29, we further evaluated its expression under hypoxic environment, and found that CYP1A2 overexpression significantly inhibited the protein level of HIF-1α along with the phosphorylated MET, AKT, and ERK, while the knockdown of CYP1A2 showed opposite effects except for ERK (Figure 6E). Therefore, the impact of CYP1A2 on HIF-1α in hypoxia appears to be identical to that in normoxia. Moreover, the interaction of the endogenous CYP1A2 and HIF-1α was demonstrated by immunoprecipitation-western blot assay in Huh7 cells (Figure 6F), and the interaction was further confirmed by the localization patterns captured by the confocal microscope under both normoxia and hypoxia (Figure 6G).

### CYP1A2 promotes ubiquitin-mediated degradation of HIF-1α protein

According to the results in Fig 6B and 6C, HIF-1α protein was down-regulated by CYP1A2 overexpression and increased after the silence of endogenous CYP1A2. However, the mRNA level of HIF-1α was not altered by either overexpression or knockdown of CYP1A2. Thus, the results indicated that CYP1A2 might weaken the stability of HIF-1α in HCC. It is known the level of HIF-1α protein is frequently affected by its stability 30. We therefore assessed whether CYP1A2 could regulate HIF-1α instability. To this end, we treated CYP1A2-overexpression or -knockdown and their scramble cells with the protein synthesis inhibitor cycloheximide. It was observed that HIF-1α was more vulnerable in the presence of CYP1A2 in PLC/PRF/5 cells but more stable in the CYP1A2-absent Huh7 cells (Figure 6H). Besides, we examined whether CYP1A2-HIF-1α interaction decreased the stability of HIF-1α by affecting its ubiquitination-related degradation. PLC/PRF/5 and Huh7 cells were treated with the proteasome inhibitor MG132 after transfecting CYP1A2 or control vectors, and then cellular lysates were immunoprecipitated with anti-HIF-1α or IgG. As speculated, CYP1A2 induced ubiquitination level of HIF-1α (anti-HIF-1α IP product) and HIF-1α expression was restored after treatment with MG132, suggesting that CYP1A2-induced HIF-1α degradation depends on the ubiquitin-proteasome system (Figure 6I).

## Discussion

Human CYPs families are generally membrane-associated proteins abundantly expressed in the liver 31, 32. The CYP family is well known for its responsibility in the metabolism of various drugs; therefore, considerable attentions have been directed to the pharmacokinetics and pharmacodynamics of CYP1A2 in cancers. A decade ago, CYP1A2 in non-tumoral liver tissues was selected as the best predictive marker for post-surgical recurrence 14. The authors further validated the predictive significance of CYP1A2 by performing a prospective multicenter study on 211 HCC patients. The CYP1A2 (-) patients had significant lower recurrence-free survival than CYP1A2 (+) patients 14. Previously, our team demonstrated that down-regulation of CYP1A2 impaired the 17β-estradiol (E2) metabolism, weakening E2-mediated tumor suppression, which contributed to HCC progression 16. However, no correlations were found between CYP1A2 and gender difference in our tissue bank (data not shown), and whether CYP1A2 influences other biological function remains obscure.

The fact of the high abundance of CYP1A2 in normal liver tissues indicates the critical role of CYP1A2 in maintaining the normal cell function in the liver 16, 33. A gradual decline in CYP1A2 level/activity has been observed from the fibrotic/cirrhotic liver to HCC, with corresponding decrease in liver function capacity 33. With this connection, we performed gain- and loss-of-function assay on CYP1A2 both *in vitro* and *in vivo*. Our data demonstrated that the reduction of CYP1A2 promoted the growth of HCC whereas its upregulation arrest the tumor progression. Emerging evidence has indicated that HGF/MET signaling plays a crucial role on tumor formation, development, maintenance, metastasis as well as the efficacy to systemic therapy, including sorafenib, in HCC 3, 6, 8, 34-36. Surprisingly, the anti-MET monotherapy, such as tivantinib, failed to show satisfactory outcomes in the phase III clinical trial 37. More experiments have been performed to explore the reason for the elusive results. A recent finding proposed the double-edged role of MET because on the one hand, it promoted carcinogenesis and metastasis while at the same time promoted anti-tumorigenic activity in cancer 38. Since both HGF and MET are significantly elevated in HCC patients, and such elevations suggest poorer prognoses 3, 6, 39, 40, it is essential to further investigate the underlying mechanism of MET and its suppressors for better understanding of HGF/MET signaling in order to develop effective approach for maximize efficacy of anti-HGF/MET treatment in HCC.

Apart from HGF-dependent MET stimulation, MET can be activated by HIF-1α. HIF-1α, a primary transcriptional factor, transcripts multiple genes to enhance proliferation, induces migration and invasion, and serves as a poor prognostic marker in HCC 41. HIF-1α is commonly active and increased in hypoxic conditions via the PI3K/AKT axis and activation of NF-κB 42. Surprisingly, our data showed that, in CYP1A2-deficient HCC cells, HIF-1α could be upregulated in an oxygenated environment. The increased synthesis of HIF-1α may further induce the MET signaling and consequently generate the positive loop of HIF-1α/MET/PI3K/AKT/NF-κB (Figure 6J). In the study, we also illustrated the inhibitory effect of CYP1A2 on HIF-1α protein expression through ubiquitin-dependent degradation (Figure 6I). The von Hippel-Lindau (VHL) tumor suppressor gene product (pVHL) is well-known for oxygen-dependent degradation of HIF-1α 43. However, the Western blot assay in the CYP1A2 abundant/absent HCC cells failed to show the interaction between CYP1A2 and pVHL. Whether there exists any potential regulation between CYP1A2 and pVHL will be an interesting topic for further investigation.

The MMP2, MMP7, and MMP9 have been demonstrated to enhance HCC cell invasiveness 44. MMP7 embraces broad substrate specificity, and cleaves basement membrane proteins, extracellular matrix, and activates proMMP2 and proMMP9 to facilitate invasive malignancy ability 45. Our experiments have shown that the expression of MMP7 in CYP1A2-deficient Huh7 and SMMC-7721 was approximately ten- and two-times higher than the scramble control, respectively, and the phenomenon of which can be reversed by CYP1A2 overexpression. Similar results have also been found on MMP2 and MMP9. It is therefore concluded that CYP1A2 can overcome MMPs-mediated tumor progression.

The HCC-inhibitory function of CYP1A2 was further supported by the clinicopathological data as its expression was inversely correlated with AFP level, vascular invasion, as well as poor TFS. AFP, a direct guidepost for auxiliary diagnosis of HCC, is not only associated with tumor size, differentiation, invasion, and metastasis but an independent risk factor of the survival of HCC patients 46, 47. Vascular invasion is a prognostic marker correlated with early HCC recurrence 48. On the contrary, it has been reported that a high MET level was identified in HCC patients with higher AFP and more frequent portal vein invasion 49.

There are limitations in our study. The CYP1A2-overexpression significant inhibited both mRNA and protein expression of MMP9 in PLC/PRF/5 cells but only suppressed the mRNA level of MMP9 in HepG2 cells. Also, the expression of MMP7 was largely different from the mRNA and protein levels in Huh7 cells. Because the post-transcriptional mechanisms are complex and varied, the phenomenon cannot be explained at the moments and need to be further investigated.

In conclusion, in normal liver, CYP1A2 is highly expressed to control the levels of HIF-1α and HGF, inhibiting MET and its downstream molecules, including AKT, ERK, and p38 (Figure 6J). In HCC, the suppression of CYP1A2 stimulates HIF-1α upregulation and HGF synthesis to activate MET/PI3K/AKT/NF-κB signaling as well as MMPs, facilitating tumor growth, migration, and invasion. Taken the previous outcome and our results together, CYP1A2 may serve as a tumor suppressor as well as a novel independent prognostic marker for patients with HCC.

## Supplementary Material

Supplementary figures and tables.

## Figures and Tables

**Table A:** IRS applied in the immunochemical analysis

Intensity positive cells (%)	Weak staining = 1 point	Moderate staining = 2 point	Strong staining = 3 point
< 10% = 1 point	IRS = 1	IRS = 2	IRS = 3
10% - 50% = 2 point	IRS = 2	IRS = 4	IRS = 6
50% - 80% = 3 point	IRS = 3	IRS = 6	IRS = 9
> 80% = 4 point	IRS = 4	IRS = 8	IRS = 12

**Table 1:** Correlation of CYP1A2 expression with clinical pathologic features in Hong Kong HCC patients (n = 90)

CYP1A2	Total	High	Low	*P*-value
Age (years)	59.0 ±9.8	58.6 ± 9.7	60.8 ± 10.2	0.52
**Gender**				*P* > 0.99
Male	75	61	14	
Female	15	12	3	
Tumor size (cm)	4.83 ± 3.52	4.78 ± 3.28	5.06 ± 4.52	0.60
Tumor number	1.33 ± 0.86	1.40 ± 0.94	1.06 ± 0.24	0.15
AFP	742.63 ± 2554.73	703.85 ± 2766.54	906.88 ± 1377.62	0.0042
ALT	45.86 ± 32.58	47.97 ± 34.97	36.8 ± 17.2	0.46
Albumin	43.73 ± 17.98	44.37 ± 19.80	41.00 ± 4.86	0.29
Bilirubin	11.66 ± 5.92	11.62 ± 6.13	11.82 ± 5.04	0.70
INR	1.05 ± 0.10	1.06 ± 0.10	1.04 ± 0.08	0.76
**HbsAg**				0.39
+	80	66	14	
-	10	7	3	
**HCV**				*P* > 0.99
+	3	3	0	
-	68	54	14	
Missing	19	16	3	
**Vascular invasion**				0.0369
Positive	20	13	7	
Negative	70	60	10	
**Cirrhosis**				0.44
Yes	59	46	13	
No	31	27	4	
**Metastasis**				0.11
Yes	19	13	6	
No	71	60	11	
**Recurrence**				0.42
Yes	45	38	7	
No	45	35	10	
**Pathologic Grade**				0.57
Well	7	7	0	
Moderate	80	63	17	
Poor	2	2	0	
Missing	1	1	0	

## Abbreviations

Ctrl: control
CYP1A2: cytochrome P450 1A2
GAPDH: glyceraldehyde 3‐phosphate dehydrogenase
HCC: hepatocellular carcinoma
HGF: hepatocyte growth factor
MET: c-mesenchymal-epithelial transition
MMP: matrix metalloproteinase
NF‐κB: nuclear factor kappa B
PCNA: proliferating cell nuclear antigen
PI3K: Phosphatidylinositol-3-kinase
