# Supplementary material for: CYP1A2 suppresses hepatocellular carcinoma through antagonizing HGF/MET signaling
Source: Theranostics. 2021 Jan 1;11(5):2123–36. doi: 10.7150/thno.49368 (PMC7797680; doi:10.7150/thno.49368)
Supplement: Supplementary file 1 — Supplementary figures and tables. [file thnov11p2123s1.pdf]

Figure S1. The mRNA of CYP1A2 is significantly decreased in TCGA-LIHC dataset from GEPIA (**P* < 0.05) (N, non-tumor tissues; T, tumor tissues).

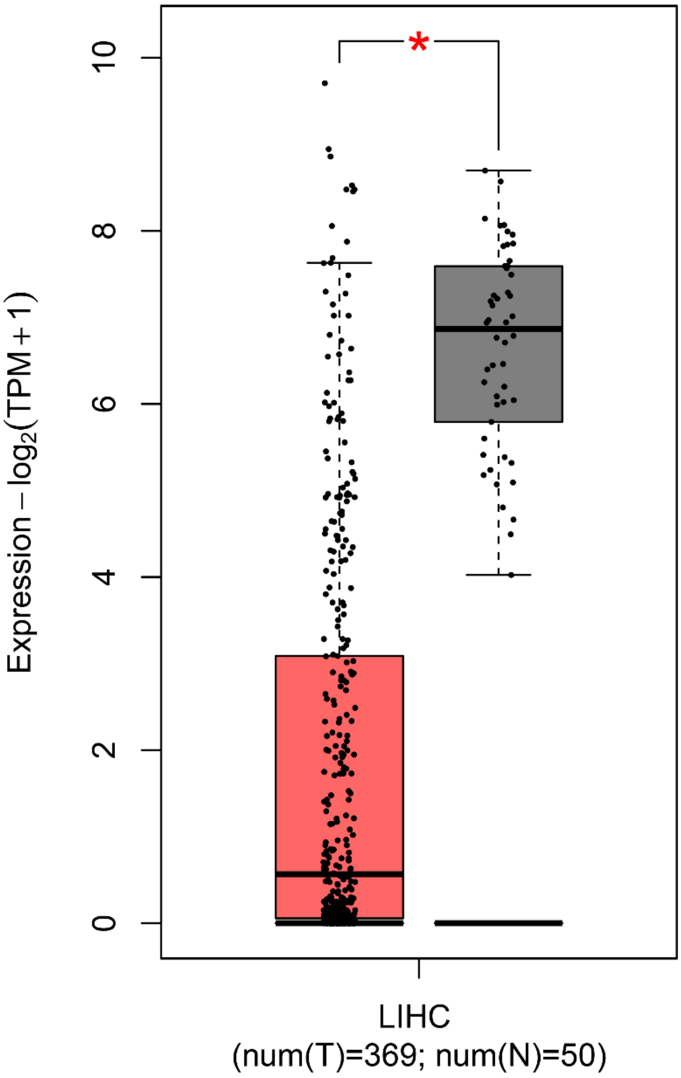

Figure S2. No significant difference was observed between the expression of CYP1A2 mRNA and the clinical pathological stage of HCC in TCGA-LIHC dataset. Stage I: n = 163; Stage II: n = 77; Stage III: n = 76; Stage IV: n = 3.

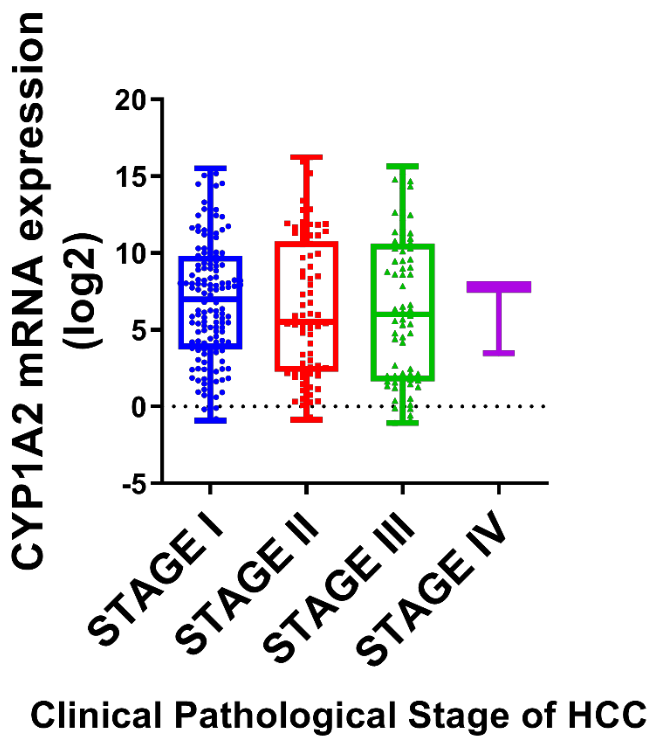

Figure S3. The HCC patients with higher expreesion of CYP1A2 displayed better survival in HCCDB online database. The data was acquired from http://lifeome.net/database/hccdb/home.html

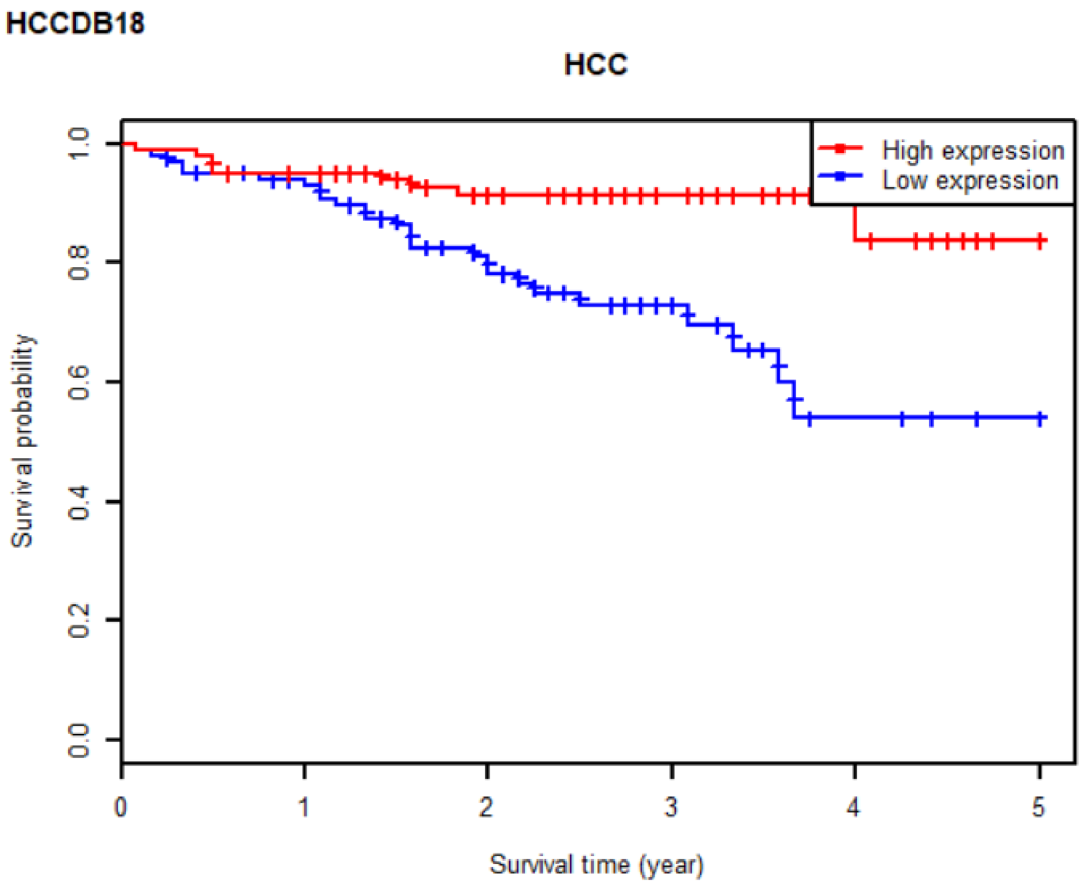

Figure S4. The endogenous expression of CYP1A2 in different HCC cell lines.

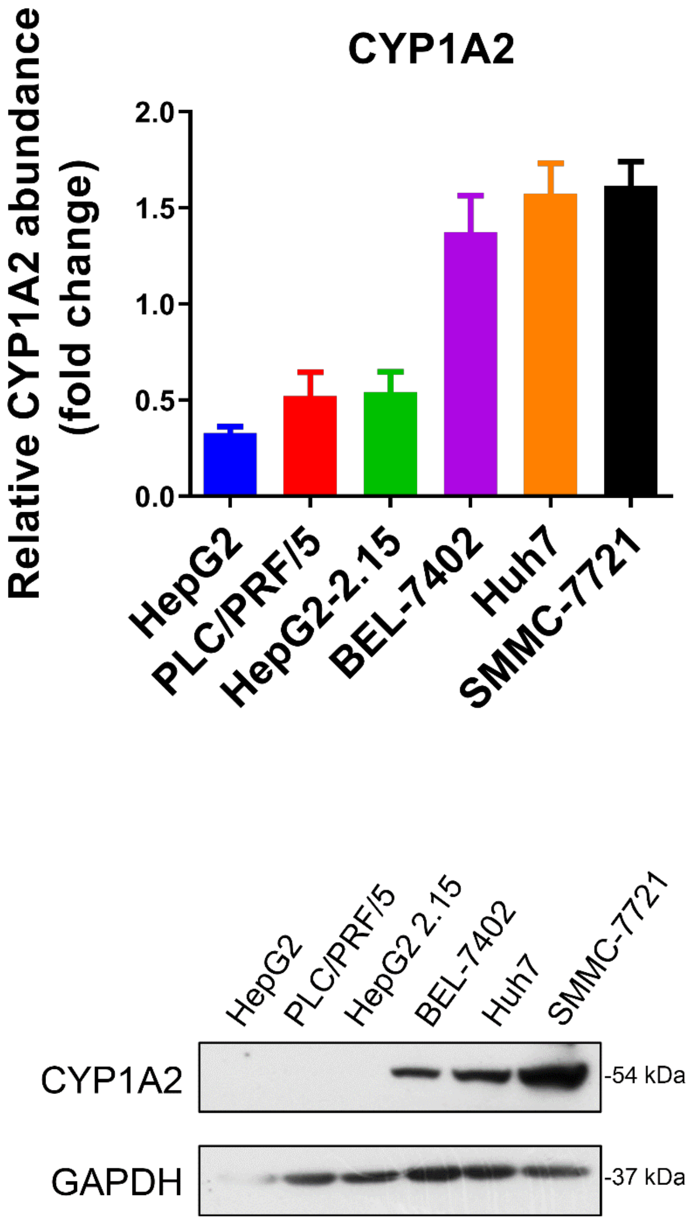

Figure S5. The expression of MET and MMPs in the TCGA-LIHC dataset (***P < 0.001).

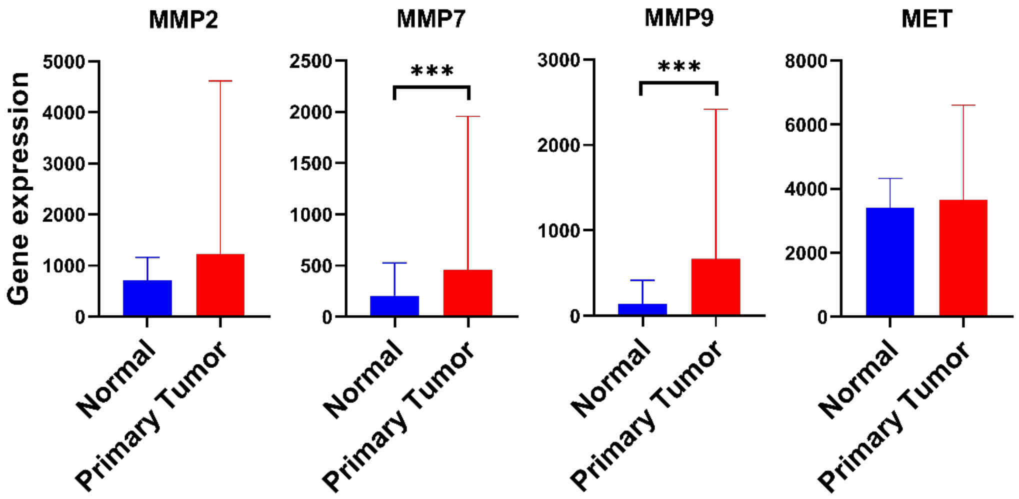

Figure S6. The effects of CYP1A2 on HGF/MET signaling after administration of HGF (40ng/ml).

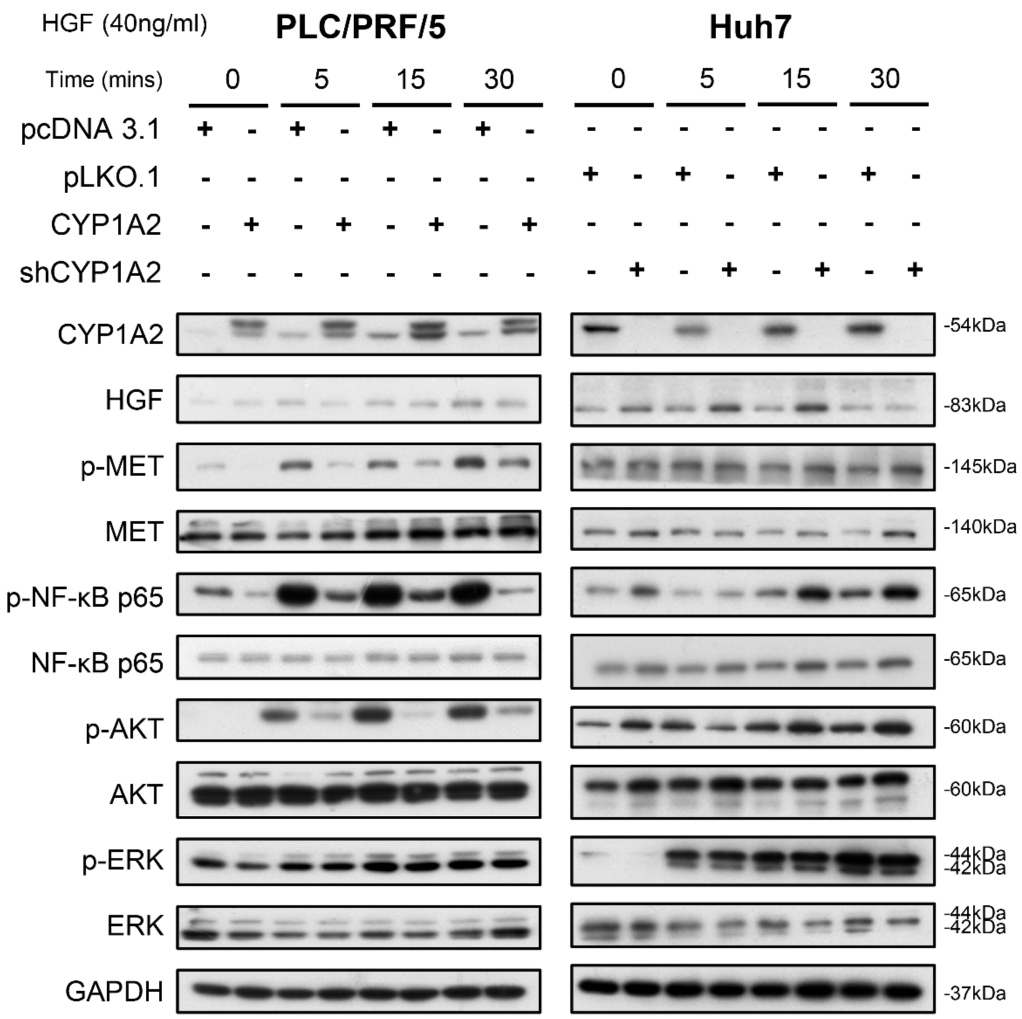

Figure S7. MTT assays showed the effect of PHA-665752 on PLC/PRF/5 and Huh7 cells at different time points. Ctrl, DMSO control.

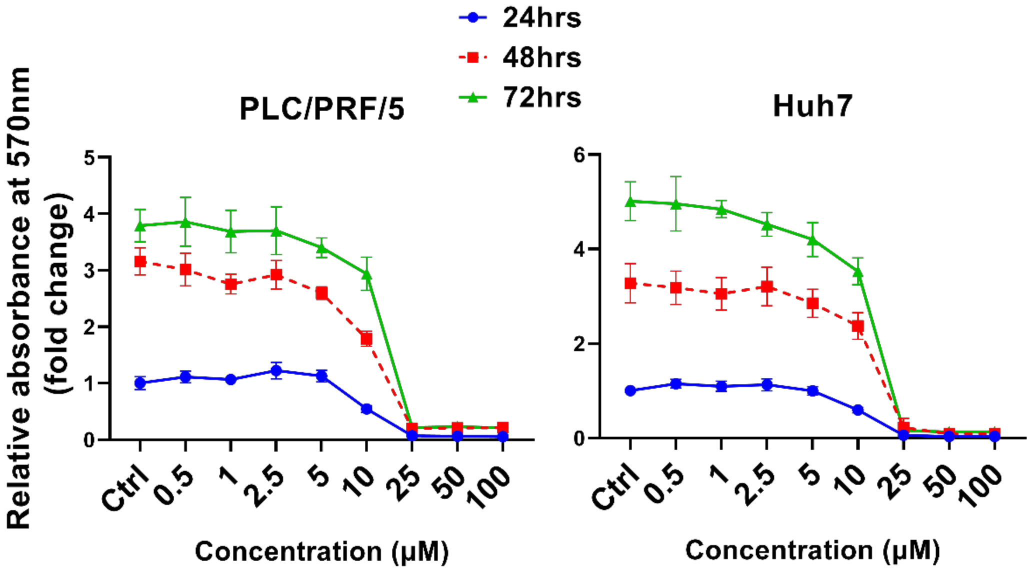

Figure S8. MET silencing inhibited the MMP2 and MMP9 expression and reversed the increased proliferation, migration, and invasion abilities in the CYP1A2-knockdown Huh7 cells. In experiment A, *** represents “pLKO.1 + N.C” verses “shCYP1A2 + N.C”.

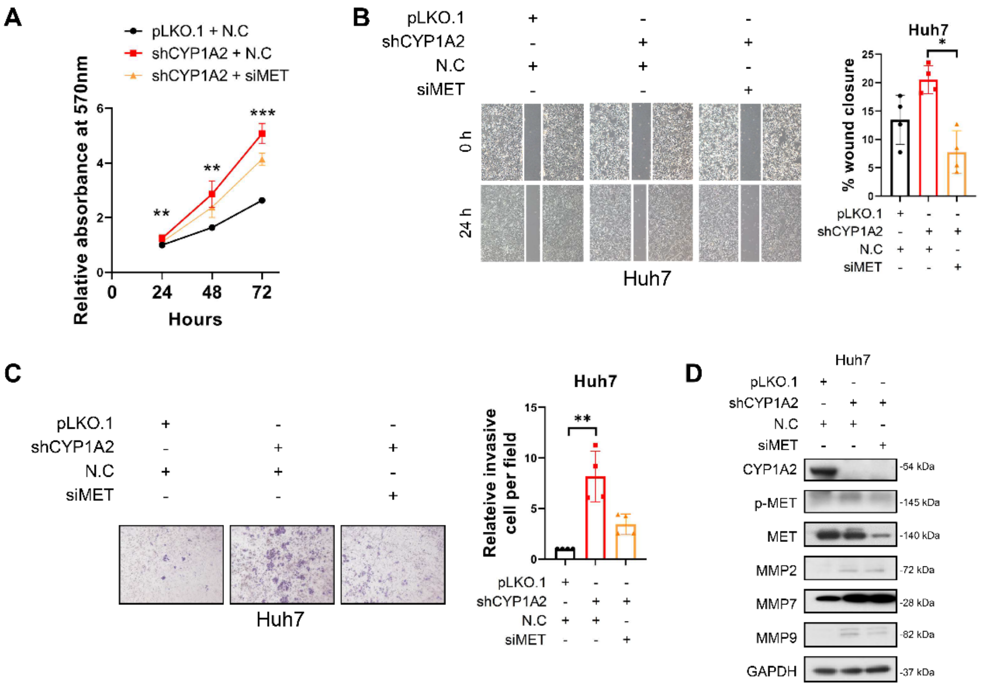

Figure S9. The positive correlation between HIF-1α-targeted gene VEGFB in Hong Kong HCC cohort and in TCGA-LIHC dataset.

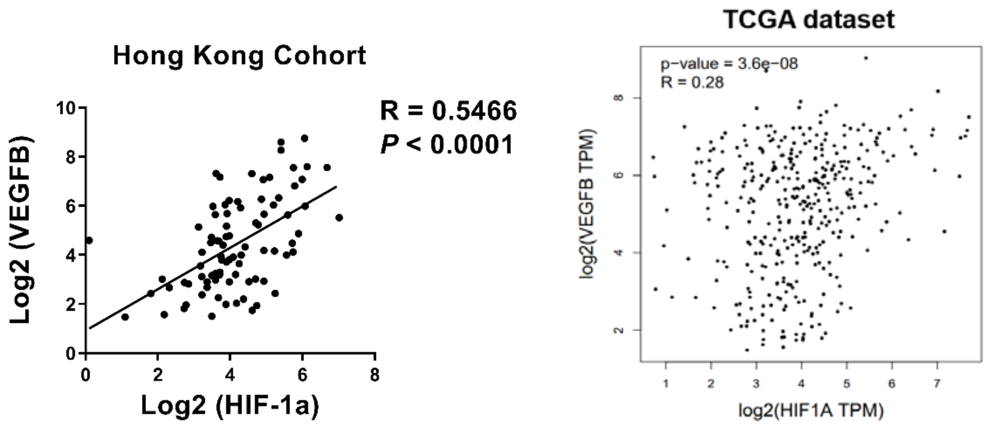

Figure S10. Western blot assay showed that LY294002 had no impact on HIF-1α expression.

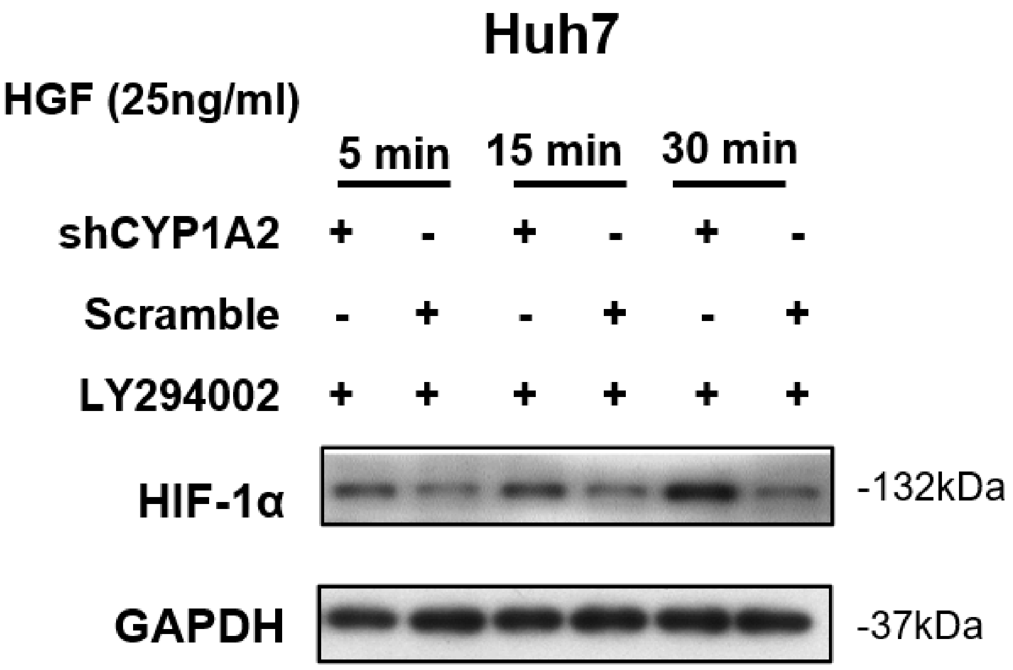

Figure S11. The five-year OS from the LIHC-TCGA dataset in accordance with CYP1A2 expression. Data were collected from https://kmplot.com/analysis/ and the patients was automatically split by the website using the Auto Select Best Cutoff.

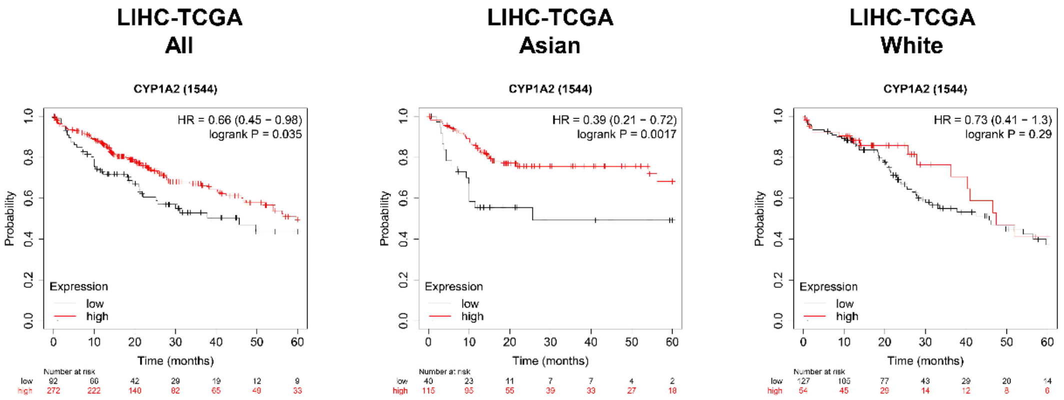

Table S1. Sequences of primers used

Primer names	Sequences (5' -> 3')
**qPCR**	
CYP1A2-F	GGACACAACGCTGAATG
CYP1A2-R	CATCATCTTCTCACTCAAGG
MMP2-F	GTCTGAAGAGCGTGAAG
MMP2-R	AGGTAGGAGTGAGAATGC
MMP7-F	GCCTACCTATAACTGGAATG
MMP7-R	AAGCCTTTGACACTAATCG
MMP9-F	CGTGACCTATGACATCC
MMP9-R	CCTCCAGAACAGAATACC
HIF-1α-F	TTCCAGTTACGTTCCTTCGATCA
HIF-1α-R	TTTGAGGACTTGCGCTTTCA
MET-F	GCACCCTAAAGCCGAAATG
MET-R	GATGACAACAGAGAAGGATACG
HGF-F	TGATACCACACGAACAC
HGF-R	AACTTCTGAACACTGAGG
GAPDH-F	CACTGGCGTCTTCACC
GAPDH-R	GAGGCTGTTGTCATACTTC
**Oligo design for shRNA cloning**	
shCYP1A2-F	CCGGCAAGGGACACAACGCTGAATGCTCGAGCATTCAGCGTTGTGTCCCTTGTTTTTG
shCYP1A2-R	AATTCAAAAACAAGGGACACAACGCTGAATGCTCGAGCATTCAGCGTTGTGTCCCTTG

Abbreviations: qPCR, real-time polymerase chain reaction

Table S2. Antibodies used in Western blot assay and Immunohistochemistry

	Company	Catalog No.
CYP1A2	Santa Cruz Biotechnology Dallas, TX	sc-53241
phospho-NF-κB p65 ser536	Cell signaling Danvers, MA	#3033
NF-κB p65	Cell signaling	#8242
N-cadherin	Cell signaling	#13116
HGF	Abcam	ab83760
GAPDH	Santa Cruz Biotechnology	sc-32233
phospho-Akt (Ser473)	Cell signaling	#4060
Akt	ImmunoWay Biotechnology Plano, TX	YT0178
phospho-Met (Tyr1234/1235)	Cell signaling	#3077
Met	Cell signaling	#8198
Phospho-p44/42 MAPK (Erk1/2)	Cell signaling	#4370
p44/42 MAPK (Erk1/2)	Cell signaling	#4695
Phospho-p38 MAPK (Thr180/Tyr182)	Cell signaling	#4511
p38 MAPK	Cell signaling	#9212
MMP2	Research And Diagnostic Systems, Minneapolis, MN	AF902
MMP7	Research And Diagnostic Systems, Minneapolis, MN	MAB9071
MMP9	Abcam	ab137867
E-cadherin	Cell signaling	#14472
N-cadherin	Cell signaling	#13116
